# Effects of Ving Tsun Chinese Martial Art Training on Upper Extremity Muscle Strength and Eye-Hand Coordination in Community-Dwelling Middle-Aged and Older Adults: A Pilot Study

**DOI:** 10.1155/2016/4013989

**Published:** 2016-07-20

**Authors:** Shirley S. M. Fong, Shamay S. M. Ng, Yoyo T. Y. Cheng, Janet Y. H. Wong, Esther Y. T. Yu, Gary C. C. Chow, Yvonne T. C. Chak, Ivy K. Y. Chan, Joni Zhang, Duncan Macfarlane, Louisa M. Y. Chung

**Affiliations:** ^1^Institute of Human Performance, The University of Hong Kong, Pokfulam, Hong Kong; ^2^School of Public Health, Li Ka Shing Faculty of Medicine, The University of Hong Kong, Pokfulam, Hong Kong; ^3^Department of Rehabilitation Sciences, The Hong Kong Polytechnic University, Hung Hom, Hong Kong; ^4^School of Nursing, Li Ka Shing Faculty of Medicine, The University of Hong Kong, Pokfulam, Hong Kong; ^5^Department of Family Medicine and Primary Care, The University of Hong Kong, Pokfulam, Hong Kong; ^6^Faculty of Liberal Arts and Social Sciences, The Hong Kong Institute of Education, Tai Po, Hong Kong; ^7^Elderly Core Business, Hong Kong Christian Service, Tsimshatsui, Hong Kong; ^8^Bliss District Elderly Community Centre, Active Ageing Service, Hong Kong Christian Service, Kwun Tong, Hong Kong; ^9^Department of Health and Physical Education, The Education University of Hong Kong, Tai Po, Hong Kong

## Abstract

*Objectives*. To evaluate the effects of Ving Tsun (VT) martial art training on the upper extremity muscle strength and eye-hand coordination of middle-aged and older adults.* Methods*. This study used a nonequivalent pretest-posttest control group design. Forty-two community-dwelling healthy adults participated in the study; 24 (mean age ± SD = 68.5 ± 6.7 years) underwent VT training for 4 weeks (a supervised VT session twice a week, plus daily home practice), and 18 (mean age ± SD = 72.0 ± 6.7 years) received no VT training and acted as controls. Shoulder and elbow isometric muscle strength and eye-hand coordination were evaluated using the Lafayette Manual Muscle Test System and a computerized finger-pointing test, respectively.* Results*. Elbow extensor peak force increased by 13.9% (*P* = 0.007) in the VT group and the time to reach peak force decreased (9.9%) differentially in the VT group compared to the control group (*P* = 0.033). For the eye-hand coordination assessment outcomes, reaction time increased by 2.9% in the VT group and decreased by 5.3% in the control group (*P* = 0.002).* Conclusions*. Four weeks of VT training could improve elbow extensor isometric peak force and the time to reach peak force but not eye-hand coordination in community-dwelling middle-aged and older adults.

## 1. Introduction

Age-associated loss of muscle strength is widely acknowledged [1, 2]. One previous study showed that muscle strength in the upper extremities starts to decline by age of 40 in both men and women [1]. This deterioration is alarming, as upper extremity muscle weakness in middle-aged and older adults is associated with physical disability in the instrumental activities of daily living, functional limitations [3], and even mortality [4].

Currently, the primary treatment strategy for maintaining or improving upper extremity muscle strength in middle-aged and older adults is exercise. In Western countries, resistance training has been advocated [5, 6], whereas in the East, traditional Chinese exercises such as Tai Chi and Qigong have been recommended as strategies to improve upper extremity muscle strength in older individuals [7, 8]. However, both resistance training and traditional Chinese exercises have their limitations. Resistance training with free weights or machines is repetitive and boring. It also may not be suitable for elderly people with joint pain or symptomatic arthritis [6]. Traditional Chinese exercises (e.g., Tai Chi and Qigong) are relatively gentle and less stressful on joints and may be more enjoyable. However, the resistance (load) may not be sufficient to induce significant improvements in muscle strength [7]. Therefore, a new type of traditional Chinese exercise—Ving Tsun (VT)—has been introduced in recent years to improve muscle strength and various other health outcomes among middle-aged and older adults [9–11]. VT training is more strenuous than Tai Chi or Qigong but gentler than resistance training. It is characterized by rapid and forceful punching and arm techniques (e.g., vertical punches with or without a sandbag/focus mitt/speed ball) and a sticking-hand exercise [9]. Therefore, VT practitioners have ample opportunities to exercise their upper limbs in a way that might improve muscle strength. However, only one cross-sectional study has shown that hand-grip strength is higher in senior VT practitioners than in no-training controls [9]. No study has examined the effect of VT training on the muscle strength of upper extremities (e.g., major muscle groups, shoulder flexors, and elbow extensors) in middle-aged and older adults.

Eye-hand coordination (EHC) is an important visual-motor function that facilitates goal-directed use of the hand in daily life, for example, reaching for objects and grasping. It requires the integrated use of eyes (visual afference), arms, hands, and fingers to produce controlled, accurate, and rapid movements [12]. Diminished EHC in the elderly can result in dependency in daily activities [13]. Previous studies have shown that EHC in the finger-pointing task declines with advancing age, specifically in the time and accuracy domains [13, 14], because aging reduces the ability to modify eye movements to meet various behavioral constraints [15]. In addition, older adults have difficulty controlling eye and hand movements concurrently [15]. To improve the daily functions of elderly adults, it is necessary to identify effective treatment strategies to strengthen their EHC. Previous studies have recommended Tai Chi training [13, 14]. However, Tai Chi may be too gentle to improve upper extremity muscle strength. We hypothesized that VT might improve both muscle strength and eye-hand coordination, because VT training requires practitioners to strike shifting targets (e.g., speed ball workout with vertical punch) frequently [16, 17]. The aim of this study was to investigate the effects of VT martial art training on upper extremity muscle strength and eye-hand coordination in middle-aged and older adults.

## 2. Methods

### 2.1. Study Design

This was a nonequivalent pretest-posttest control group pilot trial. The participants were not randomized to groups and the outcome assessors were not blinded to group assignment. Ethical approval was obtained from the Human Research Ethics Committee of the University of Hong Kong. The study was explained to each participant and written informed consent was obtained from all of the participants. All of the experimental procedures were conducted in accordance with the Declaration of Helsinki.

### 2.2. Participants

Healthy community-dwelling middle-aged and older adults were recruited from the Hong Kong Christian Service Bliss District Elderly Community Centre via convenience sampling. The inclusion criteria were as follows: (1) aged between 55 and 85 years, (2) able to ambulate independently in indoor and outdoor environments, and (3) able to follow commands and communicate with other people. The exclusion criteria were as follows: (1) unstable medical conditions (e.g., uncontrolled hypertension or symptomatic arthritis), (2) recent injuries, (3) cervical or upper limb dysfunctions, (4) visual problem or eye pathology (e.g., cataract) that may affect test performance, (5) neurological disorders (e.g., stroke, Parkinson's disease, or peripheral neuropathies of the upper extremities), (6) upper limb musculoskeletal disorders (e.g., frozen shoulder), (7) cardiopulmonary diseases (e.g., chronic obstructive pulmonary disease), (8) cognitive impairment including mild cognitive impairment, (9) previously receiving regular sports or martial arts training (e.g., Tai Chi), or (10) being too frail to practice VT.

### 2.3. Intervention

The participants in the VT group attended a 4-week Ving Tsun martial art training program in the Bliss District Elderly Community Centre, Hong Kong. They received two supervised VT training sessions per week (one hour/session) in the senior center. All of the VT training sessions were delivered by a qualified VT coach (holding a Ving Tsun Athletic Association instructor certificate). The training protocol is outlined in Table 1. This protocol was modified from a typical VT syllabus for beginners [16, 17] by an experienced physiotherapist and the VT coach. It included the basic VT stance, punching technique, dynamic footwork, and the first VT form “Siu Lim Tao” (Table 1).

To increase the amount of VT training, the participants were prescribed daily VT home exercises (excluding the VT class days) during the intervention period. The home exercises were the same as those practiced in the supervised VT training sessions (Table 1). To ensure that all of the participants complied with the home training program, the VT coach checked their VT skill proficiency at each training session. The control group received no VT training during the study period and were asked to continue their usual daily activities.

### 2.4. Test Procedures

Data collection was performed in the senior center by two registered physiotherapists and two research assistants. All of the participants were assessed one week before the commencement of the VT intervention (pretest) and again in the week after it ended (posttest). The participants in both groups underwent the following assessments.

### 2.5. Outcome Measurements

#### 2.5.1. Characteristics of the Participants

Demographic information, medical history, and exercise habits were obtained by interviewing the participants. Their physical activity level was estimated by asking him/her about the type of physical activity that s/he had most actively engaged in during a typical week within the past year. The physical activity level, in metabolic equivalent (MET) hours per week, was estimated based on the physical activity intensity, duration, frequency, and the MET value assigned to that particular activity in the Compendium of Physical Activities [18]. In addition, body weight and height were measured using a bathroom scale and a cloth measuring tape stuck on the wall, respectively. Body mass index (BMI) was then calculated using the following formula: body weight (kg)/body height (m^2^). The length of the dominant arm, defined as the distance between the acromion process and index finger, was also measured using a measuring tape. Heart rate and blood pressure at rest were measured for safety reasons.

#### 2.5.2. Upper Extremity Muscle Strength

The maximum isometric muscle strength (peak force) of the participants' dominant shoulder flexors (anterior deltoid) and elbow extensors (triceps brachii and anconeus) was assessed using the Lafayette Manual Muscle Test System (Model 01165, Lafayette Instrument Company, Lafayette, IN) with standardized manual muscle testing procedures [19] and dynamometer placements [20]. Good-to-perfect intrarater reliability (intraclass correlation: 0.84–0.97) and interrater reliability (intraclass correlation: 0.79–0.94) were reported for upper limb isometric muscle strength measurements using this method in adults [21]. These two upper limb muscle groups were selected because they are crucial for daily activities (e.g., reaching for a cup) [19] and are most commonly used during VT practice (e.g., forward vertical punch) [16, 17]. Each participant completed a familiarization trial and a testing trial of manual muscle testing in which peak force was generated for 2 to 3 seconds for each muscle group. The assessor instructed the participants to contract the muscle group being measured as hard and as fast as possible during testing. Peak force (in kg) and time to peak force (in seconds), defined as the time elapsed from the start of the test until the maximum force, have been reached [20] and were recorded and used in the analysis.

#### 2.5.3. Eye-Hand Coordination

Eye-hand coordination was assessed objectively using a finger-pointing task. Each participant sat on a chair so that the upper edge of the visual display unit (a 14-inch touch screen) (Lenovo Flex 2, Lenovo, Hong Kong) was at about eye level. The hips, knees, and ankles were at 90° of flexion with both feet flat on the floor or on a platform. At the beginning of the test, the participant's dominant hand (i.e., the hand used for writing) was placed on a touchpad (Logitech Touchpad T650, Logitech, Switzerland) that was approximately 10 cm away from the touch screen. Visual targets in the form of a ball (1.2 cm in diameter) then appeared on the screen—on the left, on the right, or in the center—in random order. The visual target appeared five times at each location (i.e., 15 targets in total). The participant was instructed to point to each target on the screen with the index finger of the dominant hand as quickly and as accurately as possible. After touching the target (indicated by its disappearance), the participant immediately placed his/her hand back in the designated starting position (touchpad). Each participant was required to repeat this finger-pointing task 15 times (15 targets) [13, 22].

Accuracy (end-point accuracy, in mm) was defined as the absolute value of the deviation (linear distance) of the participant's touch location from the center of the visual target (a ball). The accuracy value of each trial was recorded and the average accuracy value of the 15 trials was calculated and used in the analysis. A smaller value indicates greater touch accuracy. In addition, the average reaction time (in milliseconds) (i.e., the duration between the appearance of the visual target on the screen and the moment when the hand leaves the touchpad) and movement time (in milliseconds) (i.e., the duration between the moment the hand leaves the touchpad and that when the index finger touches the visual target on the screen) of the 15 trials were calculated and used for analysis [22]. The test-retest reliability of this eye-hand coordination test was found to be moderate (intraclass correlation: 0.68–0.71) in older adults [13].

### 2.6. Statistical Analyses

According to our previous studies [9, 11], the effect sizes for VT training to improve upper extremity muscle strength can range from 0.77 to 1.33. Therefore, assuming an effect size of 1.1 (large), a sample of 30 participants (15 participants per group) was necessary to achieve a statistical power of 0.8 and an alpha level of 0.05. Anticipating a 20% dropout rate [11], a minimum of 18 participants was required per group (i.e., 36 participants in total).

All of the statistical analyses were performed using SPSS 20.0 (IBM, Armonk, NY). Descriptive statistics were used to describe all of the demographic and outcome variables. Independent *t*-tests (for continuous variables) and chi-square tests (for categorical variable: sex) were conducted to compare the demographic characteristics and baseline (pretest) outcome variables between the two groups. To test the effect of VT training on each outcome measure, a two-way repeated measures analysis of covariance (ANCOVA) was performed. The within-subject factor was time and the between-subject factor was group. The relevant demographic and baseline values that showed significant between-group difference were entered as covariates. The intention-to-treat (last observation carried forward) principle was used to handle incomplete data resulting from premature intervention dropouts [23]. The two-tailed significance level was set at 5%.

## 3. Results

From September to October 2015, forty-two middle-aged and senior volunteers were screened by two physiotherapists and a social worker. All of them were eligible to participate in the study. Twenty-four participants joined the VT group and 18 joined the control group. In the posttest, 7 of the participants (16.7%) dropped out—one from the VT group (4.2%) and 6 from the control group (33.3%). The reason given for dropping out of the VT group was that the participant was unable to make the time to attend the posttest, whereas the reasons for dropping out in the control group were unknown (lost to follow-up). The overall attendance rate in the VT training sessions was 91.1%. No adverse events were reported during the VT intervention. The VT home exercise compliance rate was 95.8% with an average of 7.7 hours of self-practice per week.

The characteristics of the participants are presented in Table 2. Significant differences were found in body weight, height, and BMI between the two groups. In addition, the between-group comparisons of outcome variables at the baseline revealed that the EHC reaction time was significantly higher in the control group than in the VT group (*P* = 0.035). Therefore, the EHC baseline reaction time and BMI were treated as covariates in the subsequent two-way repeated measures ANCOVA.

All of the outcome variables are described in Table 3. For the upper extremity muscle strength outcomes, a significant time effect was found in the elbow extensor peak force (*P* = 0.007). The elbow extensor peak force increased by 13.9% in the VT group and decreased by 8.6% in the control group from pretest to posttest. The time to peak force also decreased differentially in the VT group overtime (a significant time-by-group interaction: *P* = 0.033). The elbow extensor time to peak force decreased by 9.9% in the VT group but increased by 5.3% in the control group. No significant time, group, or time-by-group interactions were noted in other muscle strength outcomes (*P* > 0.05) (Table 3).

In the EHC outcomes, a significant time effect was found in the reaction time variable only (*P* = 0.002). Surprisingly, reaction time increased by 2.9% in the VT group and decreased by 5.3% in the control group. No significant time, group, or time-by-group interactions were found in other EHC scores (*P* > 0.05) (Table 3).

## 4. Discussion

This pilot study showed that the 4-week VT training program was safe and well received by community-dwelling middle-aged and older adults. The most encouraging finding was that the VT group had a significant gain in elbow extensor peak force after short-term VT training. Our previous study also showed that VT practitioners had higher hand-grip strength than nonpractitioners, indicating VT training might improve upper extremity muscle strength [9]. We postulated that the improvement in elbow extensor muscle strength (isometric peak force) in the VT group may be related to the repeated practice of the fast and powerful vertical punch. Practicing punching a focus mitt or speed ball might be good functional-neuromuscular strength training. However, as our VT program lasted for only 4 weeks, the muscle strength improvement was very likely due to neural adaptation rather than muscle fiber adaptation [24]. Future studies may increase the duration of the VT training and measure muscle strength isokinetically at a higher speed (e.g., 240°/s).

Our results also revealed that the time required to reach peak force in the elbow extensors decreased differentially in the VT group. A previous study using electromyography to monitor the muscle activity of the major limb muscles of martial arts practitioners during striking maneuvers showed that muscles (agonists) were activated twice (double peak of muscle activity) during a single movement (e.g., elbow extension). Martial arts practitioners first contracted the limb muscles (e.g., elbow extensors) maximally to initiate the motion (first peak of muscle activity). Then some muscle fibers underwent a relaxation phase as the limb segment accelerated. Upon contact with the target, the limb muscles contracted maximally again (second peak of muscle activity) [25]. This contract-relax-contract muscle activation pattern was actually emphasized in the VT vertical punch training to improve striking force and speed [16]. It is plausible that our measurements with the dynamometer recorded the first peak of muscle activity, and thus we found that VT practitioners achieved peak force faster than the controls at posttest. Further study may instead use electromyography to assess the timing of muscle activations in the upper extremities of VT practitioners.

Despite all of these positive findings in the elbow extensors, no significant changes were noted in the shoulder flexor peak force and time to peak force in either group. We postulated that this was because when punching a target (using the VT vertical punch) the agonist (prime mover) is the triceps; the shoulder flexor (anterior deltoid) primarily acts as a stabilizing synergist to prevent shoulder extension when the triceps contract and keep the shoulder in 90° of flexion [19]. As the shoulder flexor was not the major muscle that underwent strength training during this exercise, muscle strength did not improve in the shoulder flexor.

We also found that, overall, eye-hand coordination did not improve in either group. The VT group even demonstrated deterioration in EHC reaction time compared to the control group. This result actually concurs with a previous study suggesting that well-trained martial art practitioners reacted slower to non-sport specific stimuli. This may be associated with deceased sensitivity to irrelevant sensory inputs after intensive martial art training [26]. In addition, the VT group demonstrated no improvement in EHC movement time compared to the control group. Movement time is defined as the duration between the moment the hand leaves the touchpad and the moment when the index finger touches the visual target on the screen. This parameter is a measure of the biomechanical delay required to generate sufficient muscle torque (forces) to complete the finger-pointing task [13]. VT training may not be able to shorten the biomechanical delay in generating muscle torques in the upper extremities. Another possibility is that our VT training volume or duration was not enough to induce such favorable changes.

Our results showed no significant changes in EHC end-point accuracy in either group. Previous studies reported that Tai Chi may improve EHC accuracy as the eyes often follow hand movements during training [13, 14]. This is not the case in VT training. During VT practice, the participants were encouraged to look forward while performing the arm techniques, as if they were combating an imaginary opponent [16]. Although VT participants also practiced striking a shifting target (e.g., a focus mitt) in class, the punching and striking movements may be too gross and dynamic to affect the finger-pointing task (pointing to a small stationary target). As coordination is task specific [27], perhaps the gross VT-related eye-hand coordination skill may not be carried over to the static finger-pointing task, which requires fine motor control.

The major limitation of this study is that it was not a randomized controlled trial (due to time and budget constraints)—no randomization or blinding of assessors was performed. Self-selection bias (e.g., the participants who volunteered to join the VT group may have been more motivated to exercise and this may have led to greater improvements in outcomes) and assessor bias may exist [28]. Therefore, the causal relationship between VT training and upper extremity muscle strength and eye-hand coordination cannot be confirmed. Further studies may use a randomized crossover design with a sufficient washout period to confirm the results. Another limitation is that the current VT training duration (4 weeks) may be too short to induce clinically significant changes in eye-hand coordination and upper extremity muscle strength outcomes. Hence, a further randomized controlled trial with longer VT training duration is needed to confirm the results. In addition, the participants' upper extremity functions [3] were not measured in this study. We are not sure whether the improvements in elbow extensor muscle strength of the VT group are associated with better functions in daily life. Finally, our findings can only be generalized to healthy middle-aged and older adults in the community; they may not be relevant for frail/prefrail seniors or patient populations. Further studies may explore the beneficial effects of VT training among middle-aged adults, senior people, and patient populations separately.

## 5. Conclusions

Four weeks of VT training could improve elbow extensor isometric peak force and the time to reach peak force, but not eye-hand coordination, in healthy middle-aged and older adults. Therefore, VT may be a suitable exercise for community-dwelling middle-aged and older adults, as it helps them to improve their upper extremity muscle strength at minimal cost.

## Figures and Tables

**Table 1:** Ving Tsun training protocol [16, 17].

Ving Tsun training elements	Frequency	Exercise intensity/level	Approximate duration
(i) First VT form “Siu Lim Tao” (i.e., perform various upper extremity techniques in basic VT stance)	VT class: twice per week + daily home practice (excluding the VT class days)	Light	30 min
(ii) Vertical punch in basic static VT stance		Light	10 min
(iii) Vertical punch with pivoting footwork		Moderate	5 min
(iv) Vertical punch with advancing footwork		Moderate	5 min
(v) Vertical punch with retreating footwork		Moderate	5 min
(vi) Punching a focus mitt or speed ball		Moderate	5 min

VT: Ving Tsun.

**Table 2:** Characteristics of the participants.

	Ving Tsun group (*n* = 24)	Control group (*n* = 18)	*P*
Age, years	68.5 ± 6.7	72.0 ± 6.7	0.106
Sex, *n*	6 male/18 female	1 male/17 female	0.094
Body weight, kg	60.4 ± 10.7	49.4 ± 10.1	0.002^*∗*^
Body height, cm	158.1 ± 7.6	150.7 ± 11.4	0.016^*∗*^
Body mass index, kgm^−2^	24.0 ± 3.1	21.7 ± 3.6	0.028^*∗*^
Physical activity level, MET hours per week	8.5 ± 4.9	6.6 ± 5.4	0.238
Arm length, cm	66.8 ± 4.3	64.4 ± 4.7	0.091
Resting systolic and diastolic blood pressure, mmHg	130/75	129/71	>0.05
Resting heart rate, bpm	67.5 ± 10.5	70.6 ± 13.5	0.399

Mean ± standard deviation presented unless indicated otherwise.

^*∗*^ *P* < 0.05.

**Table 3:** Outcome measurements.

	Ving Tsun group (*n* = 24)		Control group (*n* = 18)		Time effect		Group effect		Time × group effect	
	Pretest	Posttest	Pretest	Posttest	*F*	*P*	*F*	*P*	*F*	*P*
*Eye-hand coordination*										
Reaction time, ms	341.46 ± 65.25	351.36 ± 67.87	399.34 ± 95.78	378.11 ± 70.49	11.489	0.002^*∗*^	0.006	0.941	0.006	0.941
Movement time, ms	439.61 ± 109.43	458.71 ± 93.48	532.45 ± 222.43	532.93 ± 226.15	2.502	0.122	0.240	0.627	0.003	0.957
Accuracy, mm	5.19 ± 5.88	3.77 ± 1.18	4.55 ± 2.12	4.55 ± 2.13	0.421	0.520	0.001	0.978	1.148	0.291
*Shoulder flexor muscle strength*										
Peak force, kg	6.97 ± 2.58	5.82 ± 1.71	5.64 ± 1.79	4.57 ± 1.63	1.305	0.260	2.471	0.124	0.832	0.367
Time to peak force, s	2.60 ± 0.51	2.39 ± 0.65	2.25 ± 0.68	2.26 ± 0.74	0.004	0.951	2.229	0.144	0.441	0.511
*Elbow extensor muscle strength*										
Peak force, kg	4.81 ± 2.12	5.48 ± 1.99	4.44 ± 1.37	4.06 ± 1.73	8.096	0.007^*∗*^	1.223	0.276	3.226	0.080
Time to peak force, s	2.52 ± 0.52	2.27 ± 0.69	2.46 ± 0.61	2.59 ± 0.56	1.631	0.209	0.378	0.543	4.908	0.033^*∗*^

Mean ± standard deviation presented unless indicated otherwise.

^*∗*^ *P* < 0.05.

## References

[1] Metter E. J., Conwit R., Tobin J., Fozard J. L. (1997). Age-associated loss of power and strength in the upper extremities in women and men. *Journals of Gerontology—Series A Biological Sciences and Medical Sciences*.

[2] Hughes V. A., Frontera W. R., Wood M. (2001). Longitudinal muscle strength changes in older adults: influence of muscle mass, physical activity, and health. *Journals of Gerontology A: Biological Sciences and Medical Sciences*.

[3] Hairi N. N., Cumming R. G., Naganathan V. (2010). Loss of muscle strength, mass (sarcopenia), and quality (specific force) and its relationship with functional limitation and physical disability: the concord health and ageing in men project. *Journal of the American Geriatrics Society*.

[4] Newman A. B., Kupelian V., Visser M. (2006). Strength, but not muscle mass, is associated with mortality in the health, aging and body composition study cohort. *The Journals of Gerontology, Series A: Biological Sciences*.

[5] Macaluso A., De Vito G. (2004). Muscle strength, power and adaptations to resistance training in older people. *European Journal of Applied Physiology*.

[6] American College of Sports Medicine (2006). *ACSM's Guidelines for Exercise Testing and Prescription*.

[7] Fong S. S. M., Ng S. S. M., Luk W. S. (2013). Shoulder mobility, muscular strength, and quality of life in breast cancer survivors with and without Tai Chi Qigong training. *Evidence-Based Complementary and Alternative Medicine*.

[8] Mustian K. M., Katula J. A., Zhao H. (2006). A pilot study to assess the influence of Tai Chi Chuan on functional capacity among breast cancer survivors. *Journal of Supportive Oncology*.

[9] Fong S. S. M., Guo X., Cheung A. P. M. (2013). Elder Chinese martial art practitioners have higher radial bone strength, hand-grip strength, and better standing balance control. *ISRN Rehabilitation*.

[10] Fong S. S. M., Ng S. S. M., Liu K. P. Y. (2014). Musculoskeletal strength, balance performance, and self-efficacy in elderly Ving Tsun Chinese martial art practitioners: implications for fall prevention. *Evidence-Based Complementary and Alternative Medicine*.

[11] Lip R. W. T., Fong S. S. M., Ng S. S. M., Liu K. P. Y., Guo X. (2015). Effects of Ving Tsun Chinese martial art training on musculoskeletal health, balance performance, and self-efficacy in community-dwelling older adults. *Journal of Physical Therapy Science*.

[12] Crawford J. D., Medendorp W. P., Marotta J. J. (2004). Spatial transformations for eye-hand coordination. *Journal of Neurophysiology*.

[13] Kwok J. C., Hui-Chan C. W., Tsang W. W. (2010). Effects of aging and Tai Chi on finger-pointing toward stationary and moving visual targets. *Archives of Physical Medicine and Rehabilitation*.

[14] Tsang W. W. N., Kwok J. C. Y., Hui-Chan C. W. Y. (2013). Effects of aging and Tai Chi on a finger-pointing task with a choice paradigm. *Evidence-Based Complementary and Alternative Medicine*.

[15] Rand M. K., Stelmach G. E. (2011). Effects of hand termination and accuracy requirements on eye-hand coordination in older adults. *Behavioural Brain Research*.

[16] Peterson D. (2006). *Look Beyond the Pointing Finger—The Combat Philosophy of Wong Shun Leung*.

[17] Fong S. S. M. (2016). *Ving Tsun Martial Art for Health 1—Understanding Siu Lim Tao*.

[18] Ainsworth B. E., Haskell W. L., Leon A. S. (1993). Compendium of physical activities: classification of energy costs of human physical activities. *Medicine and Science in Sports and Exercise*.

[19] Kendall F. P., McCreary E. K., Provance P. G. (1993). *Muscle Testing and Function with Posture and Pain*.

[20] Lafayette Instrument (2012). *Lafayette Manual Muscle Test System User, Instructions*.

[21] Leggin B. G., Neuman R. M., Iannotti J. P., Williams G. R., Thompson E. C. (1996). Intrarater and interrater reliability of three isometric dynamometers in assessing shoulder strength. *Journal of Shoulder and Elbow Surgery*.

[22] Tsang W. W. N., Fong S. S. M., Cheng Y. T. Y. (2014). The effect of vestibular stimulation on eye-hand coordination and postural control in elite basketball players. *American Journal of Sports Science*.

[23] White I. R., Horton N. J., Carpenter J., Pocock S. J. (2011). Strategy for intention to treat analysis in randomised trials with missing outcome data. *British Medical Journal*.

[24] McArdle W. D., Katch F. I., Katch V. L. (2010). *Exercise Physiology—Nutrition, Energy, and Human Performance*.

[25] McGill S. M., Chaimberg J. D., Frost D. M., Fenwick C. M. J. (2010). Evidence of a double peak in muscle activation to enhance strike speed and force: an example with elite mixed martial arts fighters. *Journal of Strength & Conditioning Research*.

[26] Chung P., Ng G. (2012). Taekwondo training improves the neuromotor excitability and reaction of large and small muscles. *Physical Therapy in Sport*.

[27] Sailer U., Eggert T., Ditterich J., Straube A. (2000). Spatial and temporal aspects of eye-hand coordination across different tasks. *Experimental Brain Research*.

[28] Portney L. G., Watkins M. P. (2009). *Foundations of Clinical Research—Applications to Practice*.

